# Tetraethyllead and tetramethyllead – Addendum: re-evaluation of the BAT values and derivation of a BLW: Assessment Values in Biological Material – Translation of the German version from 2025

**DOI:** 10.34865/bb7800e10_2ad

**Published:** 2025-06-30

**Authors:** Annette Greiner, Hans Drexler, Andrea Hartwig, MAK Commission

**Affiliations:** 1 Friedrich-Alexander-Universität Erlangen-Nürnberg. Institute and Outpatient Clinic of Occupational, Social, and Environmental Medicine Henkestraße 9–11 91054 Erlangen Germany; 2 Institute of Applied Biosciences. Department of Food Chemistry and Toxicology. Karlsruhe Institute of Technology (KIT) Adenauerring 20a, Building 50.41 76131 Karlsruhe Germany; 3 Permanent Senate Commission for the Investigation of Health Hazards of Chemical Compounds in the Work AreaDeutsche Forschungsgemeinschaft, Kennedyallee 40, 53175 Bonn, Germany. Further information: Permanent Senate Commission for the Investigation of Health Hazards of Chemical Compounds in the Work Area | DFG

**Keywords:** Bleitetraethyl, Bleitetramethyl, Biologischer Arbeitsstoff-Toleranzwert, BAT-Wert, Biologischer Leitwert, BLW, tetraethyllead, tetramethyllead, biological tolerance value, BAT value, biological guidance value, BLW

## Abstract

The German Senate Commission for the Investigation of Health Hazards of Chemical Compounds in the Work Area (MAK Commission) re-evaluated the data for tetraethyllead [78-00-2] and tetramethyllead [75-74-1] considering all toxicological endpoints and derived a biological guidance value (BLW). Relevant studies were identified from a literature search. In 1994, the previous biological tolerance values (BAT values) for tetraethyllead and tetramethyllead were derived in correlation to the maximum workplace concentration (MAK value) of 50 µg as lead/m^3^. In 2023, the MAK value for organic lead compounds was lowered to 4 µg as lead/m^3^. There are no suitable studies for a corresponding reduction of the BAT value or the derivation of a specific BAT value for tetraethyllead. The former BAT values for tetraethyllead and tetramethyllead in urine are therefore withdrawn.

Tetraethyllead is metabolised to triethyllead, diethyllead and inorganic lead. Tetram﻿eth﻿yllead is metabolised to trimethyllead, dimethyllead and inorganic lead. Exposure to inorganic lead can best be measured via the blood lead concentration. For inorganic lead, a BAT value of 150 µg lead/l blood was derived in 2022. As organic lead compounds are more lipophilic and lead to more pronounced neurological effects than inorganic lead compounds, adverse effects cannot be excluded with certainty by compliance with the value of 150 µg lead/l blood, making a BAT value inapplicable. As a result, for tetraethyllead and tetramethyllead, a BLW of 150 µg lead/l blood is derived. Sampling time is not fixed in the steady state.

**Table:** 

**BAT value (2024)**	**not established**
**BLW (2024)**	**150 µg lead/l blood**
	Sampling time: not fixed in the steady state
	
**MAK value (2023)**	**0.004 mg as lead/m^3^**
Carcinogenicity (2024)	Category 4
Developmental toxicity (2024)	Group A
Absorption through the skin (1966)	H

## Re-evaluation

A BAT value for tetraethyllead of 25 μg diethyllead/l urine (calculated as lead) was derived by the Commission in 1994 (translated in Bolt 1995) in correlation to the MAK value of 50 μg/m^3^ (calculated as lead) valid at the time. In addition, a BAT value for total lead in urine of 50 μg/l urine was specified. For tetramethyllead, a BAT value of 50 μg total lead/l urine was established (translated in Bolt 1999). Moreover, the BAT documentations noted the necessity of compliance with the BAT value for inorganic lead in blood. In 2023, the MAK value for organic lead compounds was reduced to 4 μg as lead/m^3^ (Hartwig and MAK Commission 2024), such that the BAT value for organic lead compounds has to be re-evaluated as well.

## New studies on occupational exposure to organic lead compounds

Since publication of the last BAT documentations (Bolt 1995, 1999), a few studies on organic lead compounds have been published.

From 1991 to 1994, Kapaki et al. (1998) determined the blood lead concentration in (i) 42 gas-station employees, (ii) 47 taxi drivers, (iii) 47 bus drivers, and (iv) 36 age-matched control persons (primarily hospital workers); moreover, EEGs were recorded in some participants. No statistically significant differences in blood lead concentrations were found: (i) 56.4 ± 17 µg/l, (ii) 59.6 ± 17 µg/l, (iii) 58.8 ± 13 µg/l, and (iv) 57.6 ± 17 µg/l. Between the control group and individuals with potential occupational exposure, no differences were observed with respect to the presence of abnormal EEG findings; no significant differences were found in the blood lead levels between participants with normal versus abnormal EEG findings. Compared to the control group, however, there were differences regarding unspecific clinical symptoms (Hartwig and MAK Commission 2024; Kapaki et al. 1998).

As part of the aforementioned study by Kapaki et al. (1998), 37 gas-station employees, 41 bus drivers, and 44 taxi drivers with blood lead levels of 55 ± 16 µg/l, 58 ± 13 µg/l, and 59 ± 16 µg/l, respectively, were investigated for cortical atrophy and abnormal calcifications by computer tomography (CT). For the gas-station employees, an increased odds ratio (OR) of 6.43 (95% confidence interval (CI): 1.46–28.27) was found for cortical atrophy. Abnormal calcifications were not observed. The blood lead levels of this group, which was deemed exposed, was not increased when compared to 36 non-exposed control persons. CT scans were not performed on the control group (Varelas et al. 1999).

Afolabi et al. (1999) investigated 35 individuals who had contact with tetraethyllead, as well as 40 individuals who worked loading tanker trucks, in comparison to a control group of 36 elementary-school teachers. Lead levels were found to be 445 ± 104 µg/l blood, 513 ± 136 µg/l blood, and 346 ± 62 µg/l blood, respectively, and therefore also far above the BAT value for inorganic lead in the control group. In comparison to the control group, both groups with potential occupational exposure experienced sleep disorders, nervousness, and productive coughing with significantly higher frequency. Furthermore, the worsening of some parameters of lung function was described.

Yassin und Lubbad (2013) determined the blood lead values of 72 gas-station workers and surveyed by questionnaire the presence of 19 symptoms. The mean blood lead level was 114 μg/l. A t-test was used to investigate whether the mean blood lead levels of the participants with a certain symptom differed with statistical significance from those of participants without that symptom. This was the case with respect to irritability, headaches, concentration problems, sleep disorders, and high blood pressure. In contrast, no significant differences in the mean blood lead levels could be determined regarding fatigue, comas, cramping, seizures, loss of hearing, drop hand/drop foot, loss of libido, nausea, dyspepsia, constipation, stomach pain, gingival lead line(s), kidney pain, or infertility.

Vural and Duydu (1995) measured the urinary lead excretion of 15 gas-station employees exposed to tetraethyllead, differentiating between total lead (79.0 ± 41.1 µg lead/g creatinine) and inorganic lead (37.3 ± 33.2 µg lead/g creatinine). In contrast, a control group of 15 individuals residing 20 km from Ankara exhibited concentrations of 5.5 ± 1.6 µg lead/﻿g creatinine for total lead and 3.9 ± 1.6 µg lead/g creatinine for inorganic lead.

Further publications by Duydu et al. (1998) and Duydu and Vural (1998) investigated total lead and inorganic lead in the urine of 49 workers who filled tanks at an oil refinery as well as that of 50 motor mechanics who used gasoline to clean their hands and of 42 gas-station service employees. As a control group, 35 Ankara residents without occupational exposure were selected. The following concentrations were found: for the oil-refinery workers: 91.1 ± 37.1 µg lead/g creatinine for total lead and 65.8 ± 24.6 µg lead/g creatinine for inorganic lead; for the motor mechanics: 67.3 ± 42.8 and 48.4 ± 29.3 µg lead/g creatinine; for gas-station employees: 74.2 ± 41.1 and 53.9 ± 35.5 µg lead/g creatinine; for the control group: 50.5 ± 17.4 and 45.9 ± 14.1 µg lead/g creatinine.

In a study on 49 oil-refinery workers, 50 motor mechanics, and 35 male control persons, Duydu and Vural (1998) published additional measurements for aminolaevulinic acid (ALA) in urine; after creatinine-adjustment, a statistically significant increase in the exposed groups was observed.

Schwartz et al. (1993) investigated 222 employees of a tetraethyllead-production plant using a battery of neuropsychological tests, tests of olfactory capability and of the peripheral vibration threshold, and a questionnaire on psychiatric symptoms. In 29 exposure areas, air concentrations of 4–119 μg/m^3^ for organic lead and 1–56 μg/m^3^ for inorganic lead were estimated. Employees were divided into exposure groups based on cumulative lead exposure and exposure dura﻿tion. Associations were found between worse neuropsychological test results and higher cumulative lead exposure and exposure durations. Comparison with 62 non-exposed employees of the same facility yielded a correlation between blood lead lev﻿els and reaction times (simple visual reaction time). In the exposed group, the blood lead lev﻿els were 205 ± 100 µg/﻿l with a range of 10–510 µg/l. The employees had a mean maximum urinary concentration of 143 ± 130 µg lead/l (range 0–﻿1035 µg/l). No blood or urinary measurements were available for the comparison group. The data indicate a non-linear relationship: At blood lead levels of more than 300 μg/l, subjects exhibited, according to the authors, a considerable increase in average reaction times (Balbus et al. 1997). Another evaluation of this collective of 222 employees yielded positive correlations of age and smoking cigarettes with blood lead levels. Unexpectedly, however, alcohol consumption was associated with lower blood lead levels. In cases of increasing exposure to organic lead, workers who consumed alcohol exhibited a lower increase in blood lead levels compared to workers who did not drink alcohol. Such an effect modification could not be observed with inorganic lead compounds (McGrail et al. 1995). In a case series by Mitchell et al. (1996), 58 employees of the same company were investigated in a university occupational-health facility at their own request due to concerns about lead exposure. The employer provided information on earlier lead levels in blood and urine for 48 individuals, whereby the mean blood lead level amounted to 261 ± 91 µg/l, the mean urinary lead concentration was 51.2 ± 18.8 µg/l, and the maximum urinary lead concentration was between 36 μg/l and 500 µg/l. The current blood lead levels were 194 ± 65 µg/l on average (n = 39). The most frequently reported symptoms included memory loss, joint pain, sleep disorders, irritability, paraesthesia, fatigue, and nightmares. Due to symptoms of the peripheral nervous system or other anomalous findings in their physical examinations, the nerve conduction was measured in some patients. Polyneuropathy was diagnosed in eleven of these patients. At least one abnormality was found in 22 of the 31 patients examined (see Hartwig and MAK Commission 2024).

Several studies focused on examining individuals who have experienced mixed exposure to organic and inorganic lead in the past:

Several studies were performed on workers exposed to organic lead. In an initial study, 543 people who worked with organic lead compounds were investigated via neuropsychological testing. In this case, their last occupational exposure dated back an average of 17.8 years. To estimate earlier lead exposure, current lead concentrations from the tibia were considered. Assuming a half-life of 27 years as well as first-order elimination, tibia lead concentrations in the last year of employment were calculated. The mean value was 23.7 ± 17.4 µg lead/g bone mineral. The estimated tibia lead concentration in the last year of employment was significantly negatively associated with performance in several neuropsychological tests: Wechsler Adult Intelligence Scale – Revised Vocabulary Subtest, serial digit learning test, Rey Audito﻿ry-Verbal Learning Test (immediate recall and recognition), Trail-Making Test B, finger tapping, Purdue pegboard test (30 seconds; right hand, left hand, both hands; assembly of a pen and a ring washer), and Stroop Test (Stewart et al. 1999).

In this collective, Schwartz et al. (2000) investigated the temporal progression of cognitive performance over four years in 535 previously exposed individuals as described above as well as in 118 control persons. As an exposure measure, the current blood lead level was measured in the first year of the investigation and was correlated with the estimated tibia lead concentration. For the participants, whose occupational exposure dated back an average of 16 years, the current blood lead level was 46 ± 26 µg/l. Compared with a control group (n = 118), the lead-exposed workers performed worse in three tests (visuo-constructive abilities, verbal memory and learning) over the years. A high tibia lead level correlated with a reduction in cognitive abilities in six tests (verbal memory and learning, visual memory, executive ability, and manual dexterity) (see Hartwig and MAK Commission 2024).

In another study on 532 previously exposed persons, Schwartz et al. (2007) investigated the relationships between cognitive functions and brain volumes, which were determined using magnetic resonance imaging. The study found statistically significant correlations between lead exposure and changes to specific regions of the brain.

In another 2010 longitudinal study with participants from the same collective, no association was found between tibia lead concentrations and changes to brain volumes or lesions in the white matter over an average investigation period of five years (Schwartz et al. 2010).

Further information, including on the publications of Stewart et al. (2002, 2006), as well as a summary of Stewart and Schwartz (2007), can be found in Hartwig and MAK Commission (2024).

Leaded aviation fuel is used in some small aircraft. In two studies, higher blood lead levels were observed in children who lived near airports in North Carolina (Miranda et al. 2011) and California (Zahran et al. 2022). Zahran et al. (2022) investigated the correlation between air lead concentrations at airports and blood lead concentrations in children. According to the model calculation, an increase of 1 μg/m^3^ in the air lead concentration corresponds to an increase in blood lead level of 40.5 μg/l in children. This correlation is based on a two-month measurement of air lead levels prior to collecting blood. If the measurement of air lead levels is limited to the 30 days prior to blood collection, the estimated increase in blood lead level is reduced to 24.5 μg/l (95% CI: 9.3–39.6).

## Re-evaluation of the BAT value

Since publication of the last documentation, no studies have been published which deliver sufficiently reliable results to derive an assessment value for tetraethyllead or tetramethyllead.

For example, in some of the studies described above, no sufficient biomonitoring data were reported, and information on effects or air concentrations were often not available. Due to lead concentrations above the previously established BAT values, no updated BAT value can be derived.

In the studies of Kapaki et al. (1998) and Varelas et al. (1999), the blood lead levels in the exposed groups were not increased at the time of the investigation, compared to those of the control group. Data on earlier lead exposures were missing. Other substances, such as other fuel constituents, could not be ruled out as causes for the observed findings. As a result, these publications do not deliver any insights which would be relevant for the derivation of a limit value for lead in biological material.

Concerning the work of Yassin and Lubbard (2013) it should be borne in mind that gasoline contains, among other ingredients, various hydrocarbons. Fuel constituents, like benzene, toluene, xylene, and n‑hexane (cf. ATSDR 1995), are to be considered causes for the reported symptoms. Moreover, age as an influencing factor is a matter of concern regarding both lead levels and the frequency of some symptoms. A control group was not included in the study. It cannot be conclusively determined to what extent the statistical analysis with multiple t-tests (without correcting for multiple testing) affects the significance of the results. Health effects from organic lead compounds at blood lead levels of less than 150 μg/l can thus not be proven with sufficient certainty. Overall, no assessment value for tetraethyllead or tetramethyllead could be derived based on these studies.

In many of the cited studies, collectives with mixed exposure to organic and inorganic lead were investigated (e. g. in studies by Stewart et al. (1999), Schwartz et al. (1993) and in other investigations with this collective). This likewise applies to collectives in which lead exposure primarily originated from the use of leaded gasoline. As described in Hartwig and MAK Commission (2024), the majority of the tetraethyllead or tetramethyllead in leaded gasoline is converted to inorganic lead during the combustion process. This aspect must be taken into account, for example, in studies with taxi and bus drivers as well as in examinations on children living in the vicinity of airports. No BAT value for tetraethyllead or tetramethyllead can be derived from the studies with mixed exposures.

Even the studies on individuals previously exposed to lead cannot be used for the derivation of a BAT value as, among other data, biomonitoring results in blood or urine during the period of exposure were not given, and because these studies likewise involved mixed exposure to organic and inorganic lead.

There are therefore no new studies on organic lead compounds which would enable the derivation of a specific BAT value for tetraethyllead or tetramethyllead without correlation to the MAK value.

As, in 2023, the MAK value for organic lead compounds was reduced from 50 to 4 μg/m^3^ (as lead), the previously established BAT value for tetraethyllead was derived in correlation to the previously valid MAK value of 50 μg/m^3^, and no suitable studies for a corresponding reduction in the BAT value or the derivation of a specific BAT value for tetraethyllead are available,

**the BAT value for tetraethyllead is withdrawn.**

The comprehensive analogies between tetraethyllead and tetramethyllead were already noted in the previously published documentation on tetramethyllead, and the BAT value for total lead excretion in urine, which was evaluated for tetraethyllead, was related to mixed exposures to tetraethyllead/tetramethyllead.

**For this reason, the BAT value for tetramethyllead is also withdrawn.**

## Evaluation of a biological guidance value (BLW) in blood

Tetraethyllead is dealkylated in the body. Triethyllead, diethyllead, and inorganic lead are found in the urine (Yamamura et al. 1981). Tetramethyllead likewise is dealkylated to trimethyllead, dimethyllead, and inorganic lead (translated in Greim 2001). The best parameter for the representation of exposure to elemental and inorganic lead is the blood lead concentration. For this reason, the MAK value for inorganic lead compounds was also derived based on the BAT value in blood (translated in Hartwig and MAK Commission 2023).

The 1996 BAT documentation for tetraethyllead already noted the necessity of complying with the BAT value for inorganic lead in blood (Bolt 1995). In this regard, re-evaluation did not result in any changes.

Due to their lipophilia, organic lead compounds, like tetraethyllead and tetramethyllead, exhibited stronger neurotoxic effects than lead ions (Hartwig and MAK Commission 2024). As a result, it cannot be sufficiently proven that compliance with the BAT value derived for elemental and inorganic lead would not lead to adverse effects after exposure to tetraethyllead or tetramethyllead. Corresponding epidemiological studies on organic lead compounds are not available, so that the value of 150 μg lead/l blood in case of exposure to tetraethyllead or tetramethyllead can only be established as a BLW. For tetraethyllead and tetramethyllead, a

**BLW of 150 µg as lead/l blood is derived.**

Due to the long biological half-life of lead in humans, sampling time is not linked to a defined point in time (“not fixed in the steady state”).
